# A combination of AZD5363 and FH5363 induces lethal autophagy in transformed hepatocytes

**DOI:** 10.1038/s41419-020-02741-1

**Published:** 2020-07-17

**Authors:** Tapas Patra, Keith Meyer, Ratna B. Ray, Ranjit Ray

**Affiliations:** 1 Department of Internal Medicine, Saint Louis University, Saint Louis, MO 63104 USA; 2 Department of Pathology, Saint Louis University, Saint Louis, MO 63104 USA; 3 Department of Molecular Microbiology & Immunology, Saint Louis University, Saint Louis, MO 63104 USA

**Keywords:** Cancer, Liver cancer

## Abstract

Hepatocellular carcinoma (HCC) is one of the major causes of cancer-related death worldwide. High Akt activation and aberrant β-catenin expression contribute to HCC cell proliferation, stem cell generation, and metastasis. Several signaling pathway-specific inhibitors are in clinical trials and display different efficacies against HCC. In this study, we observed that a β-catenin inhibitor (FH535) displays antiproliferative effect on transformed human hepatocytes (THH). A combination treatment of these cells with FH535 and Akt inhibitor (AZD5363) exerted a stronger effect on cell death. Treatment of THH with AZD5363 and FH535 inhibited cell-cycle progression, enhanced autophagy marker protein expression, and autophagy-associated death, while FH535 treatment alone induced apoptosis. The use of chloroquine or z-VAD further verified these observations. Autophagy flux was evident from lowering marker proteins LAMP2, LAPTM4B, and autophagic protein expression by confocal microscopy using mCherry-EGFP-LC3 reporter construct. A combination treatment with AZD5363 and FH535 enhanced p53 expression, by modulating MDM2 activation; however, AZD5363 treatment alone restricted p53 to the nucleus by inhibiting dynamin-related protein activation. Nuclear p53 plays a crucial role for activation of autophagy by regulating the AMPK–mTOR-ULK1 pathway. Hep3B cells with null p53 did not modulate autophagy-dependent death from combination treatment. Together, our results strongly suggested that a combination treatment of Akt and β-catenin inhibitors exhibits efficient therapeutic potential for HCC.

## Introduction

HCC is the most common type of primary liver cancer and occurs most often in people with chronic liver diseases, such as metabolic disorder, alcohol consumption, and cirrhosis caused by hepatitis B virus (HBV) or hepatitis C virus (HCV) infection. Immunological deficiency in viral clearance from liver, and an interaction of viral proteins with host cellular proteins involved in signaling cascade, may promote liver disease progression^1^. Several anti-HBV medications help to slow down its ability to damage the liver. On the other hand, current anti-HCV drugs are successful in eliminating viral RNA load, although they do not prevent reinfection. In addition, eliminating HCV RNA load does not reduce the risk for HCC progression^2^. HCC in the context of nonalcoholic steatohepatitis is another risk factor for end-stage liver disease progression. Curative approaches are limited for patients diagnosed with HCC at an intermediate or advanced disease stage. Combination therapy for cancer treatment is a cornerstone modality that includes multiple therapeutic agents. This approach provides anticancer benefits like inhibiting tumor growth/ metastatic potential, reducing cancer stem cell populations, and drug resistance. A combination of anticancer drugs targeting diverse signaling pathways should enhance efficacy compared with the monotherapy approach, because they modulate various signaling pathways in a characteristically additive or synergistic manner^3^. Several signaling pathways are dysregulated in cancer cells and cause interruption in the homeostatic environments that usually contribute to the aberrant growth of the cells.

A wide range of solid and hematological malignancies show dysregulated PI3K/Akt/mTOR signaling due to mutations in multiple signaling components. Deregulation of the PI3K/Akt/mTOR pathway is increasingly implicated in HCC^4^. Akt, a serine/threonine-specific protein kinase, plays a key role in multiple cellular processes, including gene transcription and cell proliferation. AZD5363 is an Akt protein kinase inhibitor that binds to and inhibits all Akt isoforms^5^. Inhibition of Akt prevents the phosphorylation of AKT substrates that mediate cellular processes, such as cell division, apoptosis, glucose, and fatty acid metabolism. Targeting Akt is used as a therapy for a variety of human cancers.

The Wnt/β-catenin pathway is activated in several cancers, including HCC. Aberrant activation of the Wnt/β-catenin pathway has been observed in at least 30% of HCC, and roughly 20% of HCCs have mutations in the β-catenin gene. More than 50% of HCC tumors display nuclear accumulation of β-catenin, indicating that other factors may be involved^6^. β-catenin/CTNNB1 is a scaffold protein that interacts with a number of cell-associated molecules. Activation of the canonical Wnt/β-catenin pathway involves the binding of Wnt proteins to cell surface Frizzled receptors and LRP5/6 co-receptors. In the absence of Wnt proteins, much of the cellular β-catenin is bound to E-cadherin on the cell membrane. Active β-catenin translocates into the nucleus where it binds with TCF/LEF to control the expression of target genes. β-catenin targets, such as cyclin D1, c-myc, and survivin, promote cell-cycle progression and inhibit apoptosis^7,8^. We have shown that HCV-infected primary human hepatocytes exhibit high expression of β-catenin^9^. β-catenin inhibitors may be applicable for the prevention of organ fibrosis, second-line HCC prevention, and treating β-catenin-driven cancer.

The p53 tumor-suppressor protein is a crucial regulator of cell proliferation, DNA repair, and death. The loss of p53 transcriptional activity may result in uncontrolled cell proliferation, and accumulation of genomic injuries that culminate in tumor growth and dissemination. About half of liver cancer patients have mutations in the p53 gene, while the remaining harbor a defective wild‐type p53 pathway. In cancers that harbor wild-type p53, this protein is often inactivated, mainly by interactions with the murine double-minute (MDM) proteins, MDM2 and MDMX. p53 activation is one of the most attractive therapeutic strategies^10^, and several inhibitors of the p53–MDM2 interaction are under clinical trials. Nonetheless, given the distinct and cooperative function of both MDMs on p53 inactivation and the resistance of MDMX‐overexpressing cells to MDM2-only inhibitor (e.g., nutlin‐3a), represents an ideal strategy to achieve efficient p53 activation^11^.

Autophagy is a normal physiological process in the body for destruction of cells and maintenance of homeostasis^12^. The autophagic machinery can also be recruited to mediate selective tumor cell death. Sustained activation of SIRT6 resulted in autophagy-related cell death, a process that was markedly attenuated using either a pan caspase inhibitor (zVAD-fmk) or an autophagy inhibitor^13^. In cancer cells, autophagy has a dual role acting as a mechanism of tumor suppression or as an adaptive stress response to maintain tumor cell survival. Lethal autophagy is associated with eukaryotic cell death and apoptosis. Autophagy plays a dual role, acting as a mechanism for anticancer activity or at a basal level for cell survival by restoring homeostasis. An excessive stimulation of autophagy can be lethal for cancer cells either due to excess elimination of intracellular organelles and cytoplasmic content or activation of the Na^+^/K^+^ ATPase pump to change membrane osmolality and ion transport^12,14^. Several studies suggested that treatment with some of the inhibitors results in induction of autophagy, leading to cell death of HCC^15,16^. The clinical lysosomotropic autophagy inhibitor chloroquine cooperates with the AKT inhibitor AZD5363 to inhibit autophagy, and sensitizes tumor cells to AZD5363-induced cell death in prostate cancer xenograft models, offering as a potential therapeutic approach in the clinic^17^. Thus, components of the autophagy machinery are selectively utilized or repurposed for cell death. In this study, multiple drugs were examined for lethal effect on representative transformed hepatocytes. We observed that a combination of Akt and β-catenin inhibitors enhances antiproliferative efficacy of HCC cell lines, and may be used as potential therapeutic modalities against HCC.

## Results

### Combination treatment of FH535 and AZD5363 exhibits stronger antiproliferative effect on THH

**Fig. 1:**
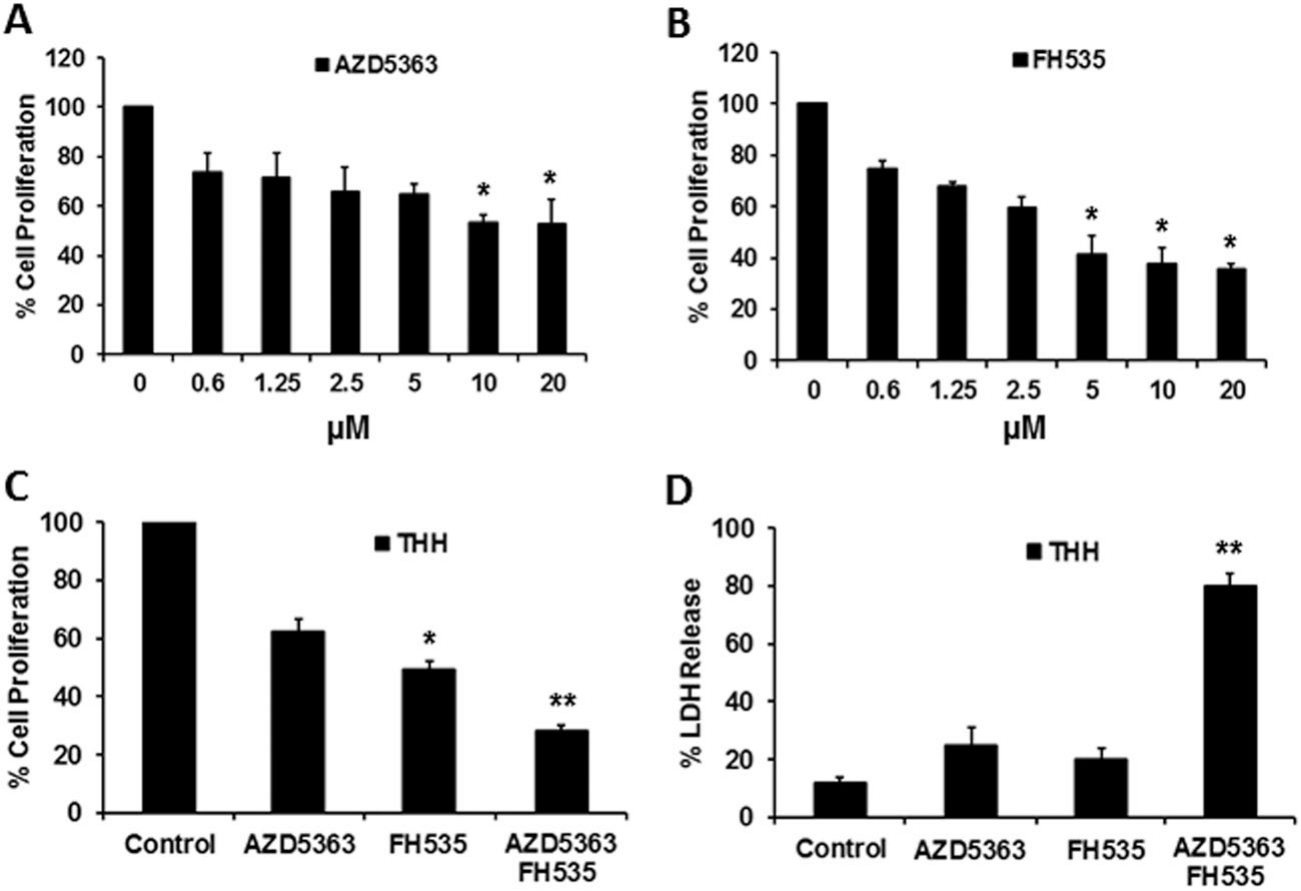
Inhibition of cell proliferation by signaling pathway-specific inhibitors. THH proliferation was analyzed by MTS assay after treatment with FH535 (**a**) and AZD5363 (**b**), at different concentrations for 72 h. Proliferation of THH was analyzed separately by MTS assay after treatment with 5 µM FH535 and/or 5 µM AZD5363 for 72 h (**c**). Cell death was examined for LDH release after treatment with 5 µM FH535 and/or 5 µM AZD5363 for 72 h (**d**). The results are presented as the mean ± SD from three independent experiments. *P < 0.05, **P < 0.005 were regarded as significant.

Many different signaling pathways contribute to the development and cellular homeostasis in cancer. Sorafenib is the first line of treatment for HCC approved by FDA, although only 30% of HCC patients benefit from sorafenib, and an acquired resistance often appears within 6 months^18^. We have used Akt inhibitor (AZD5363) and β-catenin inhibitor (FH535) at exponential doses to determine antiproliferative effect on THH. MTS assay for cell proliferation was performed with different concentrations of these signaling pathway inhibitors. Our results suggested a high efficacy of FH535, displaying a better inhibitory effect on THH growth at 72 h when compared with AZD5363 (Fig. 1a, b) or when tested with the other inhibitors (Lin28 inhibitor—C1632, Raf kinase inhibitor—sorafenib, mTOR inhibitor—AZD8055, and Stat3 inhibitor—Stattic—data not shown). We further examined a combination of these two inhibitors at multiple doses on cell growth. A combination of 5 µM AZD5363 and 5 µM FH535 treatment exhibited optimal results and an additive antiproliferative effect on THH (Fig. 1c).

Leakage of LDH from cell cytoplasm in the culture medium is an indicator of cell death. We also performed LDH-release assay to measure cell death following treatment with the inhibitors on adherent THH. Our results indicated that a combination of AZD5363 and FH535 treatment displays a much greater (~80%) cell death, while individual AZD5363 or FH535 treatment caused ~25% and ~20% cell death, respectively (Fig. 1d).

### Combination treatment of AZD5363 and FH535 accelerates autophagy- associated cell death

**Fig. 2:**
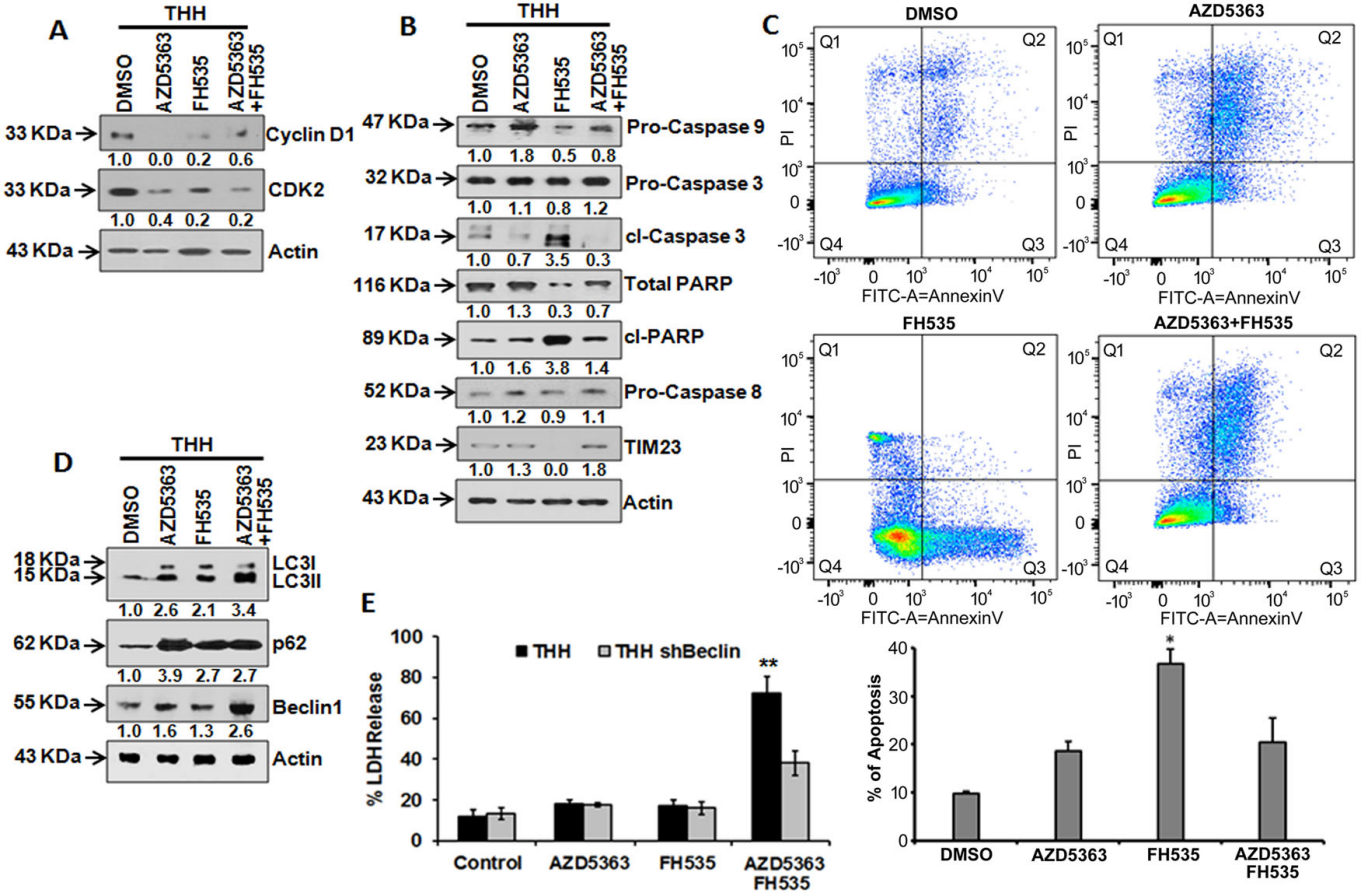
Induction of autophagy-associated proteins in THH upon FH535 and/or AZD5363 treatment. Western blot analysis was performed after treatment of THH with individual or a combination of 5 µM FH535 and/or 5 µM AZD5363 for 48 h. The expression status of cyclin D1 and CDK2 (**a**), and pro-caspase 9, pro-caspase 3, cl-caspase 3, total PARP, cl-PARP, pro-caspase 8, and TIM23 (**b**) are shown. Expression level of actin in each lane was considered for comparison of protein load and illustrated by representative blots shown at the bottom. Densitometry scanning results are shown below for each lane that normalized to the actin content and was expressed relative to controls set at 1.0. Apoptosis analysis was performed by Annexin V/PI staining after treatment of THH with individual or a combination of 5 µM FH535 and/or 5 µM AZD5363 for 48 h and detected by flow cytometry in Q3 gate (**c**). The results presented the mean ± SD of three independent experiments. *P < 0.05 was regarded as significant. Western blot analysis for expression of LC3, p62, or Beclin1 was performed after treatment of THH with individual or a combination of 5 µM FH535 and/or 5 µM AZD5363 for 48 h (**d**). Expression level of actin in each lane was considered for comparison of protein load and illustrated by representative blots shown at the bottom. Densitometry scanning results are shown below for each lane that normalized to the actin content and expressed relative to controls set at 1.0. THH and THH-harboring shRNA for Beclin1 knockdown were treated similarly with the inhibitors for 72 h and analyzed LDH-release assay (**e**). The results presented the mean ± SD of three independent experiments. **P < 0.005 was regarded as significant.

AZD5363 or FH535 treatment of THH reduced the expression of cyclin D1 and CDK2 (Fig. 2a). The results indicated that treatment with both inhibitors affected the expression of proteins associated with cell-cycle progression. Since we observed higher LDH release in combination therapy, we examined whether mono- or combination therapy induces apoptotic cell death. We observed a reduction of the level of pro-caspase 9 only upon FH535 treatment (Fig. 2b). Accumulation of cleaved caspase 3, cleaved PARP, and reduction in total PARP was also observed in FH535- treated, but not in AZD5363-treated THH, while combined treatment displayed only a modest increase in PARP status (Fig. 2b). The status of pro-caspase 8 did not alter after treatment (Fig. 2b), suggesting that the external caspase system is not activated under these experimental conditions. TIM23 is one of the inner membrane protein complexes of mitochondria, which may direct proteins across and into a membrane. TIM23 transports precursor proteins into two different mitochondrial subcompartments, the matrix and the inner membrane. Mitochondrial dysfunction is closely associated with apoptosis. Damage of mitochondrial membrane proteins occurs at an early stage of apoptosis. We observed a loss of TIM23 in solitary treatment with FH535, but not in AZD5363, or in a combination of FH535 and AZD5363 treatment (Fig. 2b). We performed flow cytometry analysis of THH following treatment with AZD5363, FH535, or in combination after Annexin V/PI staining. Accumulation of Annexin V-stained cells occurred in Q3 gate, indicating that FH535 treatment increased apoptosis as compared with others (Fig. 2c). These results suggested that both the compounds inhibit cell-cycle progression, while FH535 alone may induce apoptotic cell death.

Here, we focused on the potential for autophagy-mediated cell death in the absence of apoptosis upon treatment of THH with the combination of AZD5363 and FH535. The expression of autophagy markers, LC3, p62, and Beclin1, was examined by western blot analysis. Individual or combined treatment of THH with the two inhibitors enhanced LC3, p62, and Beclin1 expression (Fig. 2d). Beclin1 and LC3II were also expressed highely in combined treatment with AZD5363 and FH535, than induced by single inhibitors. The combination treatment failed to sensitize substantial cell death in shRNA-mediated Beclin1-depleted THH (Fig. 2e). These results demonstrated that a combined treatment of AZD5363 and FH535 exhibits stronger autophagy and that could be lethal to transformed human hepatocytes.

**Fig. 3:**
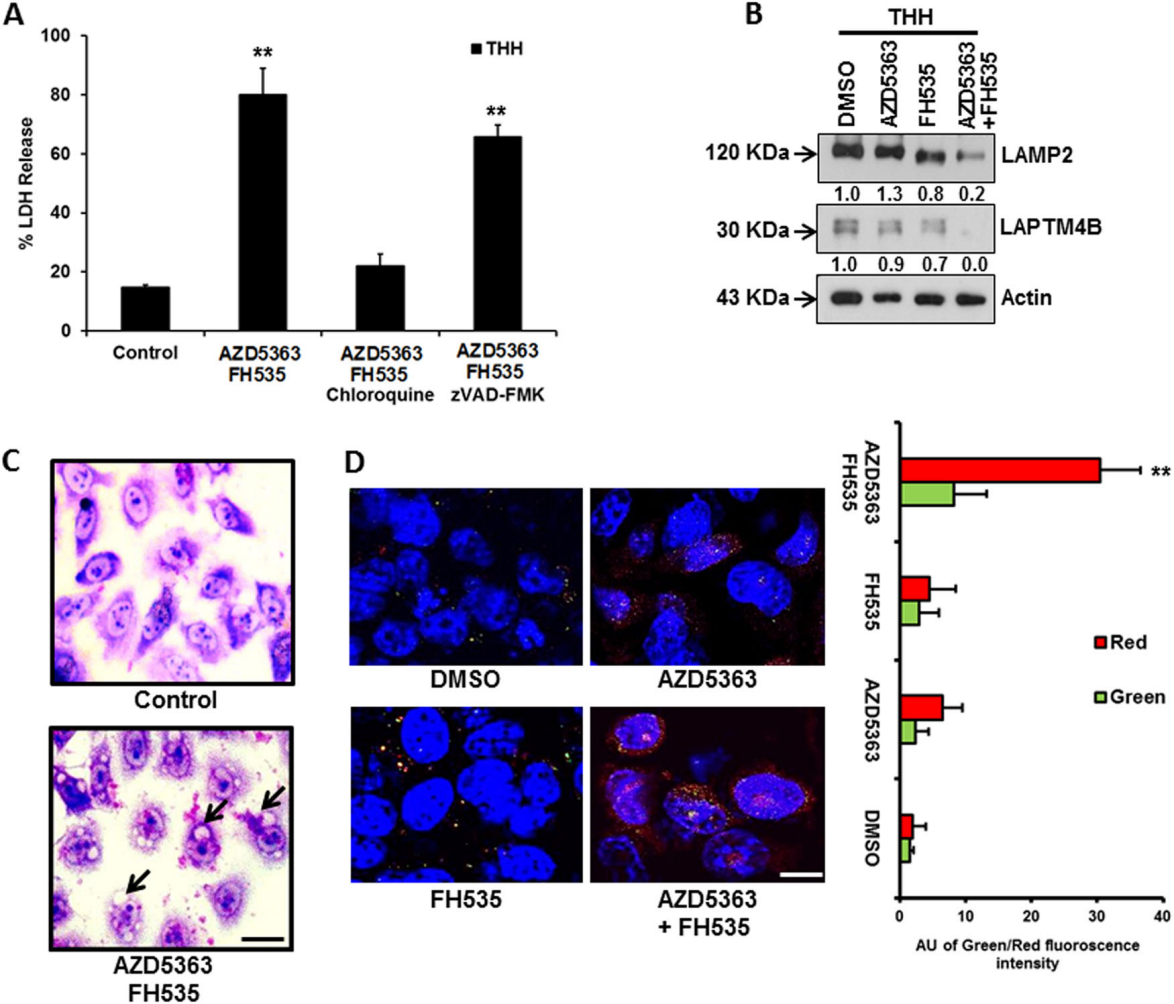
Combination of AZD5363 and FH535 promotes autophagy- mediated cell death in THH. Cellular death was analyzed from LDH-release assay at 72 h after combined treatment of THH with 5 µM AZD5363 and 5 µM FH535 in the presence of 25 µM chloroquine or 50 µM z-VAD-fmk (**a**). The results are presented as the mean ± SD from three independent experiments. **P < 0.005 was regarded as significant. Western blot analysis was performed after treatment of THH with individual or a combination of 5 µM AZD5363 and 5 µM FH535 inhibitors for 48 h. The expression status of LAMP2 and LAPTM4B is shown (**b**). Expression level of actin in each lane was considered for comparison of protein load and illustrated by representative blots shown at the bottom. Densitometry scanning results are shown below for each lane and normalized to the actin content, and are expressed as relative to controls set at 1.0. Cytoplasmic vacuole formation in THH is shown by Giemsa staining without inhibitor treatment or combined treatment with 5 µM AZD5363 and 5 µM FH535 (**c**). Scale bar indicates 10 µm. Autophagy flux was further analyzed by confocal microscopy with mCherry-EGFP-LC3 construct for red fluorescence (high autophagy flux) or green (low autophagy flux) following combination of the two inhibitor treatments (**d**). Scale bar indicates 10 µm. The results are presented as mean ± SD from three independent experiments. **P < 0.005 was regarded as significant.

Next, we analyzed the role of these two inhibitors on THH in the presence of autophagy and apoptosis inhibitors at their prior known optimum concentrations. Autophagy inhibitor chloroquine significantly inhibited AZD5363-/FH535-mediated cell death (~60%), and was almost like LDH release from untreated control, unlike the results following use of apoptosis inhibitor zVAD-fmk (Fig. 3a). The cytotoxicity blocked by the presence of the autophagy inhibitor indicated that a combination of these two inhibitors effectively reduces cell viability via an autophagy-dependent pathway.

Lysosomal marker LAMP2 and LAPTM4B were also analyzed, and interestingly, exposure of cells to a combination of AZD5363 and FH535 almost completely inhibited LAMP2 expression (Fig. 3b). May–Grünwald–Giemsa staining of THH treated with a combination of these two inhibitors revealed a massive vacuolation of cells over time of incubation, as compared with untreated control (Fig. 3c). Autophagy flux was also evaluated by confocal microscopy with mCherry-EGFP-LC3 construct in the presence or absence of the inhibitors. Confocal microscopy suggested strong autophagy flux from the appearance of red color because of a combination of the two inhibitor treatments and from red color intensity for autophagy flux (Fig. 3d). Thus, the appearance of autophagic characteristics and autophagic flux was evident from these sets of results.

### Combination of AZD5363 and FH535 modulates the MDM2–p53 axis

**Fig. 4:**
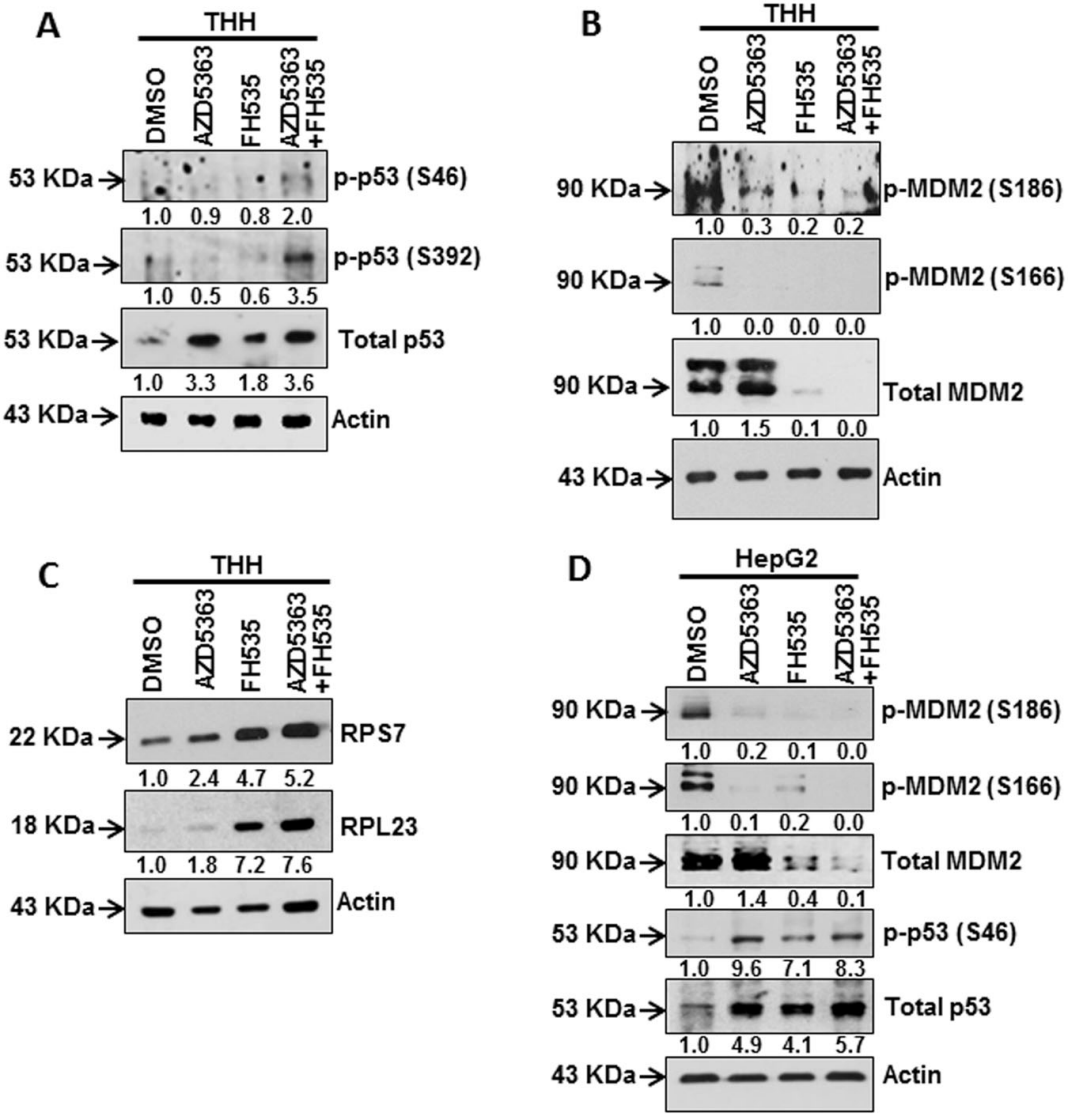
Modulation of MDM2–p53 signaling axis-related proteins in THH upon FH535 and/or AZD5363 treatment. Western blot analysis was performed after treatment of THH with individual or a combination of 5 µM FH535 and 5 µM AZD5363 inhibitors for 48 h. The expression status of phospho-p53 (Ser46, Ser392) and total p53 (**a**), phospho-MDM2 (Ser186), phospho-MDM2 (Ser166) and total MDM2 (**b**), and RPS7 and RPL23 (**c**), and the expression status of phospho-MDM2 (Ser186, Ser166), total MDM2, phospho-p53 (Ser46), and total p53 are shown from HepG2 cells (**d**). Proteins from cell lysates were estimated by Lowry reagent, and equal protein amounts were loaded in each lane to analyze actin expression status of Fig. 2d and reused here in panel **a**. Expression level of actin in each lane was considered for comparison of protein load and illustrated by representative blots shown at the bottom. Densitometry scanning results are shown below of each lane that normalized to the actin content, and was expressed relative to controls set at 1.0. Densitometric scanning of the established 90-kDa MDM2 polypeptide band was used for analysis.

p53 is a well-characterized tumor-suppressor protein and is critical for cellular functions, such as metabolism, cellular differentiation, cell-cycle regulation, apoptosis, and autophagy. Here, we examined the regulatory role of p53 in association with AZD5363 and/or FH535 treatment in facilitating an antiproliferative effect. Our results suggested that AZD5363 primarily increased total p53 level (Fig. 4a). Combined treatment with these two cell-signaling pathway inhibitors did not further increase in total p53 expression in THH. However, an induction of phosphorylated p53 at Ser46 and Ser392 residues was observed upon combination treatment. Phosphorylation at Ser392 is known to stabilize p53 tetramer formation and increased nuclear accumulation^19^, while Ser46 phosphorylation induced its transactivation for tumor-suppressor activity^20^.

MDM2 is one of the major regulators for functional activation of p53. We performed Western blot analysis for MDM2 activation in the presence of these inhibitors. Total MDM2 expression was enhanced upon AZD5363 treatment, while suppressed following FH535 treatment (Fig. 4b). Phosphorylation of MDM2 at Ser186 or Ser166 was suppressed upon AZD5363 and/or FH535 treatment of THH. Inhibition of β-catenin signaling leads to ribosomal stress responsible for induction of ribosomal proteins (RP) S7 and L23, and the degradation of MDM2^21,22^. Our further analysis suggested that FH535 treatment causes upregulation of RPS7 and RPL23 in THH, but not upon AZD5363 treatment (Fig. 4c). Treatment with these inhibitors alone or in combination displayed a similar effect on MDM2 and p53 in HepG2 cells (Fig. 4d). Therefore, our study suggested that treatment of transformed hepatocytes with FH535 significantly decreased MDM2, and markedly increased RPS7 and RPL23.

### p53 defect fails to trigger autophagy-associated protein expression and efficient cell death upon FH535 and AZD5363 treatment

**Fig. 5:**
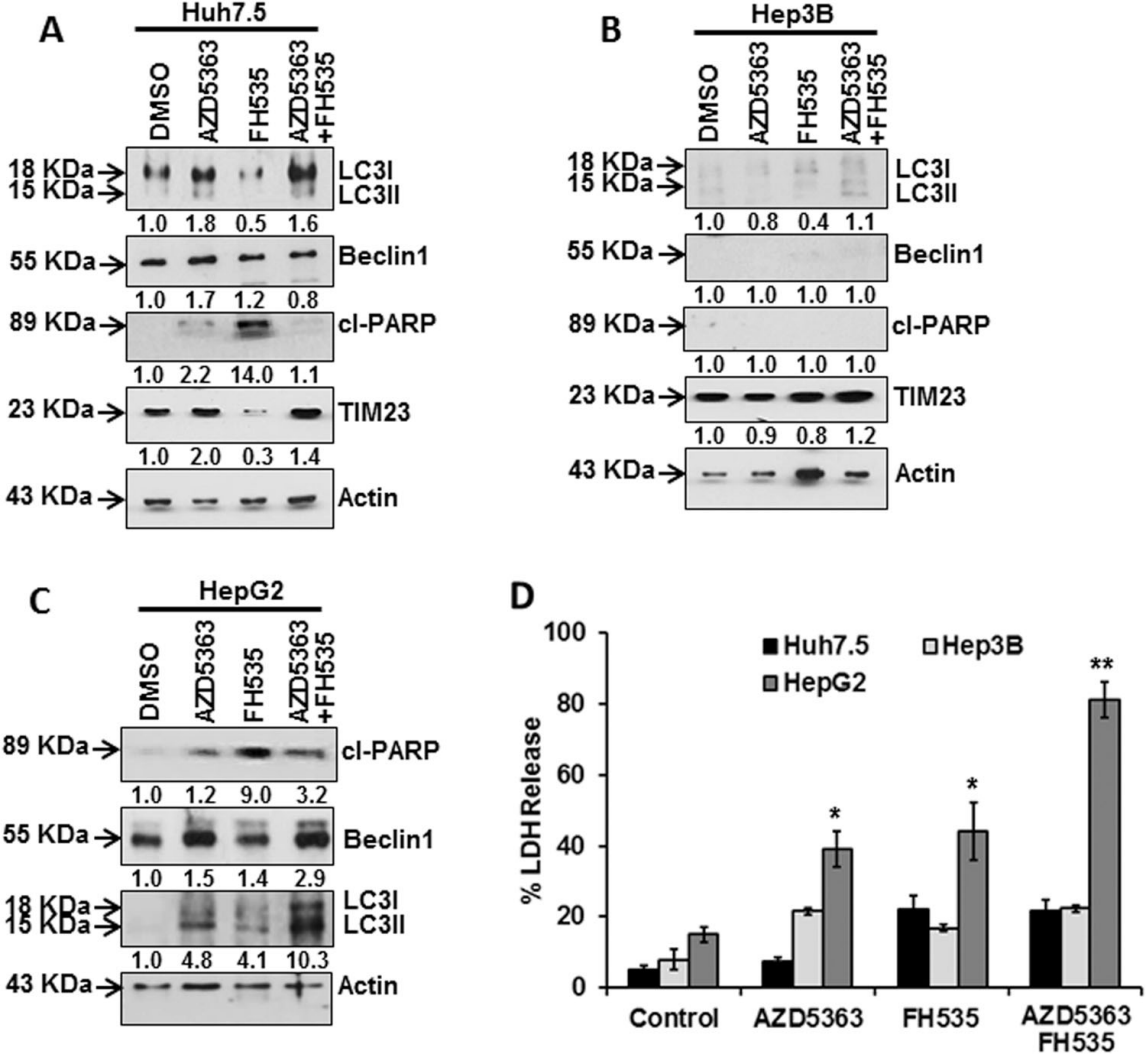
Autophagy marker expression status in etiologically different transformed human hepatocytes. Western blot analysis for expression status of LC3, Beclin1, cl-PARP, and TIM23 after treatment for 48 h with individual or a combination of 5 µM FH535 and 5 µM AZD5363 of Huh7.5 (**a**) and Hep3B cells (**b**). The expression status of cl-PARP, Beclin1, and LC3 is shown in similar inhibitor-treated HepG2 cells (**c**). Expression level of actin in each lane is shown for comparison of protein load in the blot. Densitometry scanning results are shown below for each lane that normalized to the actin content, and expressed relative to controls set at 1.0. Huh7.5, Hep3B, and HepG2 cell death were analyzed for LDH release at 72 h after individual or combined treatment of 5 µM FH535 and 5 µM AZD5363 (**d**). The results are presented as the mean ± SD from three independent experiments. *P < 0.05, **P < 0.005 were regarded as significant.

We next examined how p53 plays a regulatory function in FH535- and AZD5363-associated autophagy. Huh7.5 cells express mutant p53 (Y220C), which impairs its DNA-binding ability, whereas Hep3B cells are p53-negative, and HepG2 cells express wild-type p53^23^. We observed that LC3 increases in AZD5363-treated and AZD5363/FH535-treated Huh7.5 cells, while there was a lack in Beclin1 induction. A defect in LC3 and Beclin1 expression was clear in Hep3B cells (Fig. 5b). FH535 treatment did not enhance the autophagy-related proteins in Huh7.5 or Hep3B cells, and instead increased cleaved PARP accumulation (Fig. 5a, c). Interestingly, degradation of mitochondrial membrane protein TIM23 was also observed in FH535-treated Huh7.5 cells, but not in Hep3B cells in Huh7.5 and HepG2 cells (Fig. 5a, b). We did not observe a significant LDH release or cell death in Huh7.5 or Hep3B cells, but HepG2 cells were considerably sensitized for cell death upon a similar dose of mono- or combination treatment with FH535 and AZD5363 (Fig. 5d).

In addition, we observed that a combination treatment of FH535 and AZD5363 exhibits a significant level of Hep3B cell death following transfection with wild-type p53 gene (Supplementary Fig. 1A). The p53-transfected Hep3B cells resulted in the induction of LC3II and Beclin1 expression, without PARP alteration, upon combined inhibitor treatment (Supplementary Fig. 1B). Next, we examined the combination effect of FH535 in the presence of an autophagy-inducible inhibitor in THH. We also used an mTOR inhibitor (AZD8055), known to induce autophagy by unaltering p53 expression upon FH535-combined treatment, which did not cause substantial cell death in THH (Supplementary Fig. 1C, D). Our results suggested that the presence of wild-type p53 induces autophagy-mediated cell death, while null p53 does not. Thus, p53 appears to cause tumor cell death by these inhibitory compounds inducing autophagy.

Furthermore, autophagy inhibitor chloroquine significantly inhibited AZD5363/FH535-mediated cell death in HepG2 cells where wild-type p53 was expressed, but the presence of apoptosis inhibitor zVAD-fmk was unable to suppress AZD5363/FH535-mediated cell death (Supplementary Fig. 2A). We analyzed cell death by LDH-release assay after treatment of human kidney epithelial cells 293T, representing as control non-tumor cells, with these two inhibitors. 293T cells were not sensitized to cell death upon a similar dose of mono- or combination treatment with FH535 and AZD5363 (Supplementary Fig. 2B).

### p53 status correlates with enhanced SESN2 expression and AMPK–mTOR activation

**Fig. 6:**
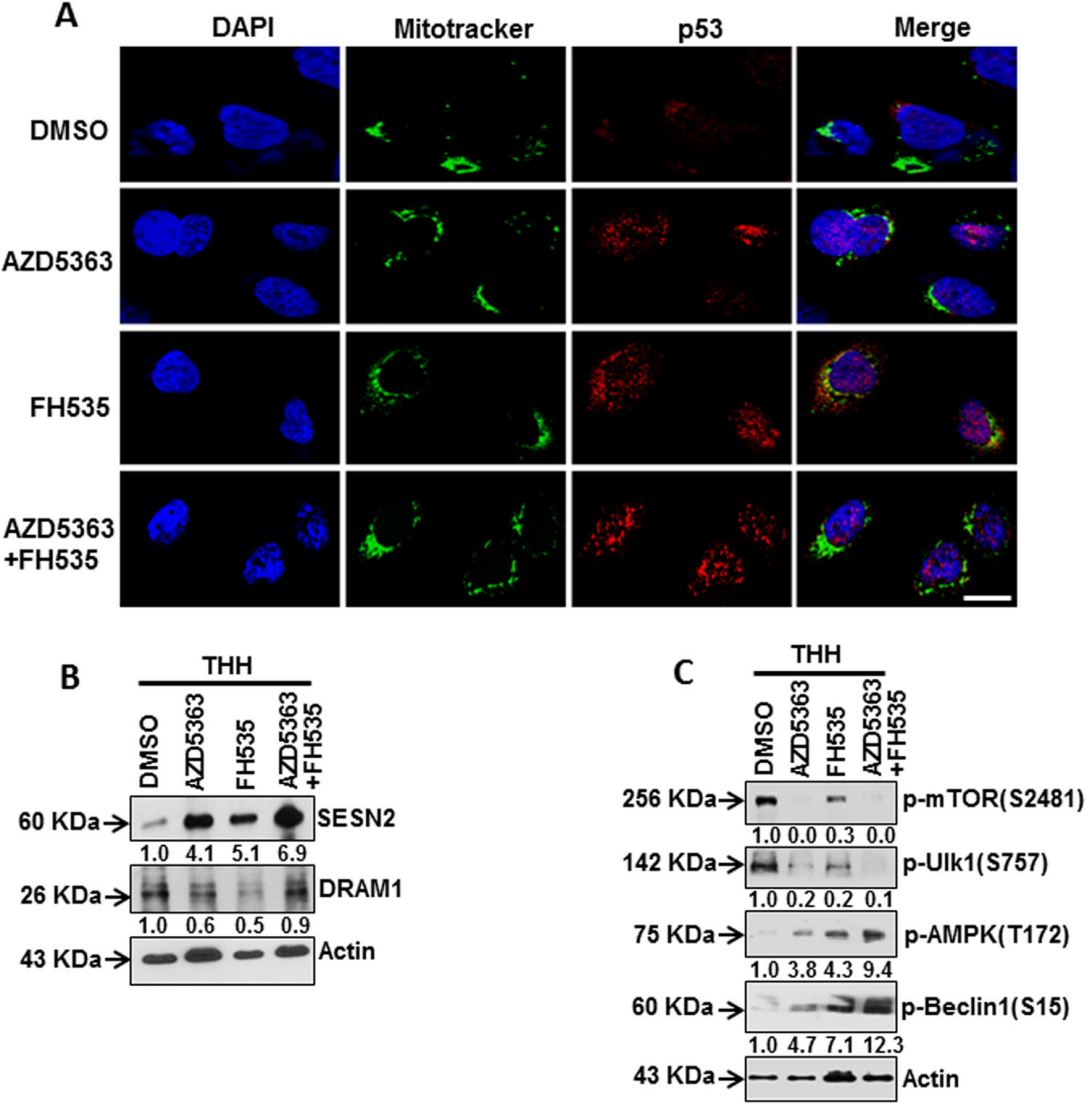
Subcellular localization of p53 and modulation of the AMPK–mTOR pathway. p53 localization was analyzed by confocal microscopy after individual or combined treatment of THH with 5 µM FH535 and 5 µM AZD5363 for 48 h. p53 stained as red, nucleus as blue (DAPI), and mitochondria as green (mitotracker) are shown (**a**). Scale bar indicates 10 µm. Western blot analysis was performed after similar inhibitor treatment of THH for 48 h. The expression status of SESN2 and DRAM1 (**b**), phospho-mTOR (Ser2481), phospho-Ulk1 (Ser757), phospho-AMPK (Thr172), and phospho-Beclin1 (Ser15) is shown (**c**). Expression level of actin in each lane is shown for comparison of protein load in the blot. Densitometry scanning results are shown below of each lane that normalized to the actin content and was expressed relative to controls set at 1.0.

Cellular stress increases p53 level, and most of it translocates into the nucleus to promote autophagy by transactivating target genes. On the other hand, p53 partially translocates to the mitochondria leading to the onset of apoptosis^24,25^. We observed that treatment of THH with AZD5363 exhibited p53 induction into the nucleus, while FH535 treatment showed both nuclear and mitochondrial localization of p53 (Fig. 6a). However, a combination of both the inhibitors reduced mitochondrial localization, and primarily enhanced nuclear p53 in THH. The nuclear localization of p53 promotes activation of stress-responsive Sestrin gene, like SESN2, and has a protective effect on physiological/pathological states mainly via regulating oxidative stress, endoplasmic reticulum stress, autophagy, metabolism, and inflammation^26^. Upregulated SESN2 induces AMPK activation and suppresses mTOR activation to promote autophagy^27,28^. Treatment with individual or combination of the inhibitors exhibited SESN2 induction by Western blot analysis from THH (Fig. 6b). DNA damage -regulated autophagy modulator 1 (DRAM1) is another transcriptional target of p53 and promotes autophagy-associated cell death^29^. However, we did not observe a change in DRAM1 expression upon combination treatment of THH (Fig. 6b). A decreased mTOR activation (phosphorylation at Ser2481) and enhanced phospho-AMPK (Thr172) levels were observed in THH upon treatment with these inhibitors (Fig. 6c). ULK1, also known as ATG1, is a key serine/threonine protein kinase acting upstream step of autophagosome formation^30^. Knockout of ULK1 results in a severe defect in the autophagy pathway. ULK1 is a target of the TOR kinase signaling pathway that regulates autophagy. AMPK, activated during low nutrient conditions, directly phosphorylates ULK1 at multiple sites. Conversely, mTOR, which is a regulator of cell growth and is an inhibitor of autophagy, phosphorylates ULK1 at Ser757 and disrupts the interaction between ULK1 and AMPK^31^. Here, we observed a decrease in phosphorylated ULK1 at Ser757 in THH-expressing p53 upon treatment with these inhibitors (Fig. 6c). Active ULK1 phosphorylates Beclin1 at Ser15 to initiate the autophagosome formation. We observed enhanced phospho-Beclin1 (Ser15) levels in THH upon treatment with these inhibitors (Fig. 6c). In addition, we observed that treatment with individual or combination of the inhibitors exhibited SESN2 induction, activation of AMPK (phosphorylation at Thr172), and a reduction of phosphorylated ULK1 (Ser757) in HepG2 cells expressing wild-type p53 (Supplementary Fig. 2C). On the other hand, Huh7.5 cells carrying mutant p53 were unable to induce SESN2–AMPK axis upon inhibitor treatment (Supplementary Fig. 2D). Further, a reduction of LAMP2 and LAPTM4B lysosomal marker expression in HepG2 cells upon combination treatment suggested autophagy- related cell death (Supplementary Fig. 2E). Therefore, our results suggested that a combined treatment of FH535 and AZD5363 is associated with enhanced accumulation of nuclear p53, and a transactivating ability for SESN2 expression, followed by modulation of the AMPK–mTOR–ULK1 signaling axis to promote autophagy in transformed hepatocytes.

### AZD5363 treatment restricts p53 nuclear localization by inhibiting dynamin activation

**Fig. 7:**
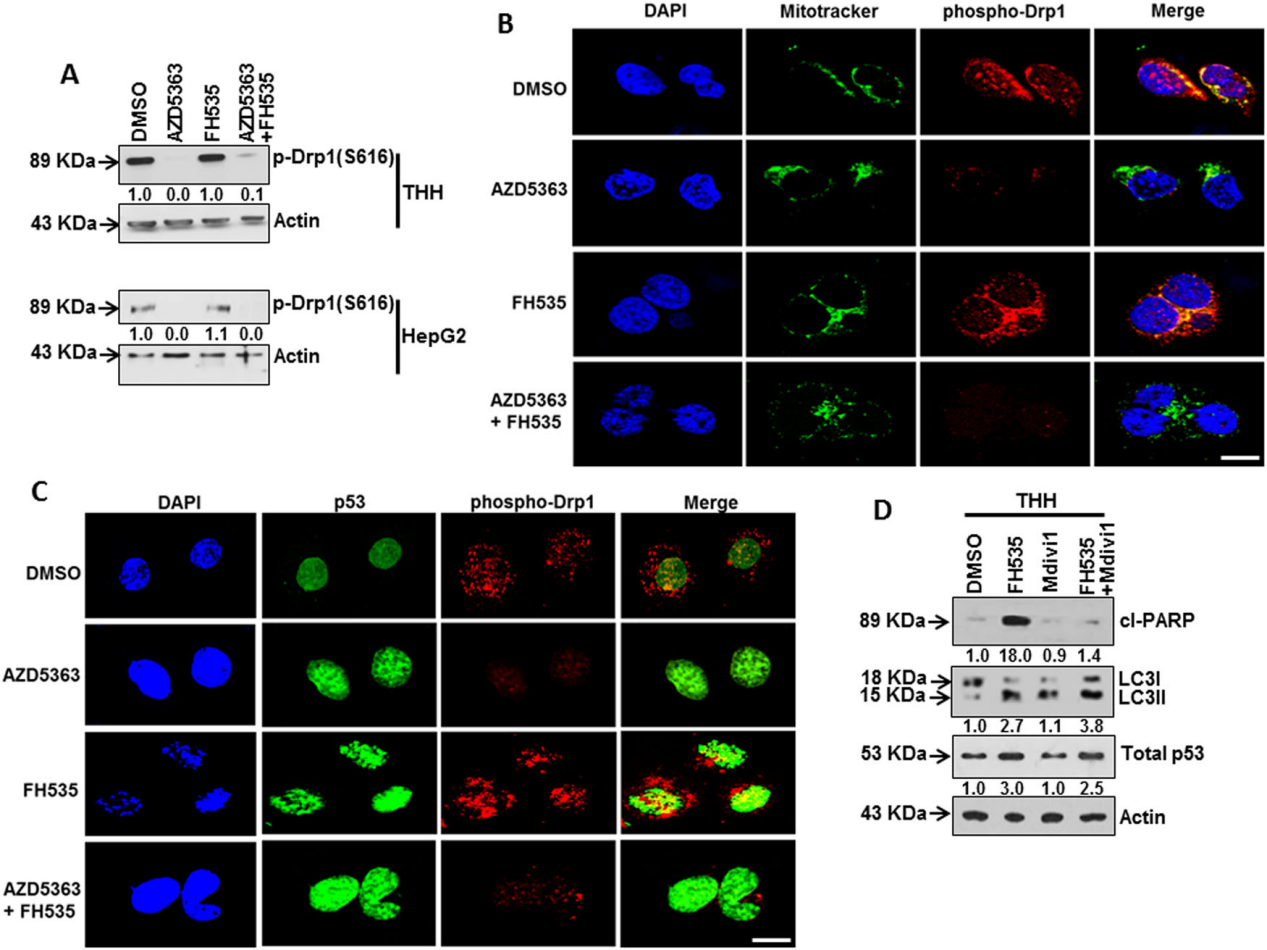
Drp1 regulation and subcellular localization of p53. Western blot analysis for phospho-Drp1 (Ser616) was performed at 48 h after individual or combined treatment of THH or HepG2 cells with 5 µM AZD5363 and/or 5 µM FH535 (**a**). Proteins from cell lysates were estimated by Lowry reagent, and equal protein amounts were loaded in each lane to analyze actin expression status of Fig. 5c and reused here in panel **a**. Expression level of actin in each lane is shown for comparison of protein load in the blot. Densitometry scanning results are shown below for each lane that normalized to the actin content and was expressed relative to controls set at 1.0. Subcellular localization of phospho-Drp1 in THH was analyzed by confocal microscopy at 48 h after individual or combined treatment of the same inhibitors. Phospho-Drp1 (Ser616) stained as red, nucleus as blue (DAPI), and mitochondria as green (mitotracker) are shown (**b**). Scale bar indicates 10 µm. Co-localization of phospho-Drp1 and p53 in THH was analyzed by confocal microscopy at 48 h after individual or combined inhibitor treatment. Phospho-Drp1 (Ser616) stained red and p53-stained green are shown at a similar magnification (**c**). Scale bar indicates 10 µm. Western blot analysis for expression of cl-PARP, LC3, and total p53 was performed after 5 µM FH535 and/or 5 µM Mdivi1 treatment of THH (**d**). Expression level of actin in each lane is shown for comparison of protein load in the blot. Densitometry scanning results are shown below for each lane that normalized to the actin content and was expressed relative to controls set at 1.0.

Dynamin-related protein 1 (Drp1), a primary mitochondrial fission protein, is required for p53 translocation to the mitochondria under stressful conditions^32,33^, and phosphorylation of Drp1 at Ser616 residue is important for mitochondrial localization^34^ for regulation of apoptotic events under stressful conditions^33,35^. We analyzed the phosphorylation status of Drp1 at Ser616 residue to evaluate the potential regulatory role of Drp1 following inhibitor treatment. The expression of phospho-Drp1 was suppressed in AZD5363-treated THH and HepG2 cells, but was not affected by FH535 treatment (Fig. 7a). Inhibition of activated Akt attenuates phosphorylation of Drp1 at Ser616^36^. Immunofluorescence analysis demonstrated that phosphorylated Drp1 translocates into the mitochondria, whereas AZD5363 treatment impaired expression of phospho-Drp1 (Fig. 7b). Further, both Drp1 and p53 co-localize in FH535-treated THH (Fig. 7c). The use of Drp1 inhibitor Mdivi1 in FH535-treated THH exhibited reduced cleaved PARP accumulation, followed by increased LC3II and unaltered p53 expression for autophagy induction in cells (Fig. 7d). Thus, treatment with AZD5363 appeared to restrict p53 to the nucleus by impairing Drp1.

## Discussion

In this study, we targeted the β-catenin and Akt -signaling pathways with small-molecule inhibitors to prevent transformed human hepatocyte growth. We have used FH535, a synthetic inhibitor of the β-catenin/TCF4-signaling axis and known to exhibit antiproliferative and anti-angiogenic effects on various carcinomas^37^, and AZD5363 as an Akt inhibitor for inhibition of cell survival. Constitutively, active Akt has been reported to protect cells from cell death^38^, and the Akt signaling axis plays an important role in HCC development^4^. Capivasertiv (AZD5363), a potent pan-Akt kinase inhibitor, is currently under investigation in Phase I clinical trial, and inhibits in vivo tumor growth^5^. Transcriptome analysis of HCC clinical specimens identified that the β-catenin and Akt signaling pathways are associated with distinct clinical outcomes^39^. Therefore, aiming to inhibit these triggered signaling modules could have potential clinical implications. Our results suggested that a combination of AZD5363 and FH535 treatment displays improved antiproliferative efficacy on THH as compared with monotherapy, and induces autophagic death, not apoptosis, since chloroquine/z-VAD-fmk treatment helped in separating these two mechanistic aspects. These inhibitors are known to work to different extents in a preclinical animal model and in clinical trials for solid tumors. FH535 inhibits the growth of xenograft tumors in mice^40^. Clinical trial of AZD5363 in combination therapy also suggested a protective role against different tumors. Thus, additional clinical studies with targeted combination therapy against β-catenin and Akt may provide valuable information.

Our present study suggested that an individual treatment of AZD5363 or FH535 induces autophagy markers in THH, but does not cause significant cell death. Interestingly, a combination of AZD5363 and FH535 treatment of THH exhibited enhanced autophagy protein expression and correlated with a significant level of cell death. We also observed that combination treatment failed to sensitize substantial cell death in Beclin1-depleted transformed hepatocytes. An excessive stimulation of autophagy can be lethal for cancer cells with different apoptotic thresholds and genetic background^13^. However, only FH535-treated cells displayed a weak modulation of caspase 9, caspase 3, and PARP for apoptosis, while the combination of AZD5363 and FH535 failed to induce apoptosis. Excessive autophagy can attenuate apoptotic cell death by selectively reducing proapoptotic proteins in the cytosol^41^. Reduction of specific proteins is due to ubiquitination by the interaction of sequestosome 1 (SQSTM1) or p62^42^. Our results supported that a high level of p62 is expressed in cells treated with AZD5363 and FH535 alone or in combination, and may be associated with a loss of apoptotic cell death.

Our study showed a combinatorial effect resulting from accumulation of induced p53 in the nucleus. However, activation of p53 contributes to promotion of cell death, while active p53 can also effectively block cell-cycle progression by inducing transcription of cyclin-dependent kinase inhibitor p21. Both functions of p53, either induction of cell death or inhibition of cell-cycle progression, can emphatically generate tumor-suppressive effect^43^. p53 controls cell death depending upon its subcellular localization. In the nucleus, active p53 can bind to the promoter region of target genes involved in cell death^44^. On the other hand, cytoplasmic p53 can relocalize to the mitochondria and trigger mitochondrial membrane permeabilization followed by apoptotic death^25^. In this study, we found that Drp1 phosphorylation representing its activated state correlated with mitochondrial translocation of p53, and resulting in FH535-induced apoptosis marker expression. This result was verified by using an inhibitor. Our results also suggested that treatment with AZD5363 individually or in combination with FH535 will suppress the phosphorylation of Drp1 at Ser616, and prevent translocation of p53 to the mitochondria.

p53 also plays a vital role in the regulation of autophagy. Nuclear p53 stimulates autophagy by transactivation, and cytoplasmic p53 inhibits the autophagic pathway^45^. We observed that a combination treatment with the two inhibitors triggers an increase in total p53 and its phosphorylated active moiety, and enhanced p53 localization to the nucleus, leading to transactivation of autophagic genes. These observations agree with the impairment in triggering lethal autophagy in combination with FH535 and AZD5363. mTOR inhibitor is well-known for inducing autophagy^46^. In our experiment, mTOR inhibitor (AZD8055) in combination with FH535 did not cause significant THH death. This further suggested that wild-type p53 facilitates cell death in our combination therapy. Therefore, activation of wild-type p53 appeared to require FH535, and AZD5363 induced autophagy-dependent cell death. In normal physiological conditions, p53 levels are controlled by MDM2-mediated ubiquitination and proteosomal degradation^47^. We observed that AZD5363 treatment did not affect the level of total MDM2, but downregulated its phosphorylated status. Inhibition of the Akt signaling pathway may attenuate activation of MDM2 because Akt directly phosphorylates MDM2^48^. Interestingly, FH535 treatment alone exhibited a decrease in total MDM2. However, ribosomal stress marker proteins RPS7 and RPL23 were upregulated by FH535 treatment, implicating that degradation of MDM2 is probably occurring due to ribosomal stress. Our results suggested that blockade of both β-catenin and Akt signaling from combined treatment results in attenuation of MDM2 for enhancement of p53 activation.

Nuclear p53 controls autophagy by transactivation of several signaling molecules. p53 binds to the promoter region of SESN2 and transcriptionally induces SESN2 expression, while upregulated SESN2 promotes activation of AMPK and attenuates mTOR to induce autophagy by ULK1^27,28^. Our results suggested that FH535 and AZD5363 treatment induces SESN2 expression followed by modulation of AMPK–mTOR–ULK1 signaling. Inhibitor-treated nuclear localization of induced p53 regulates autophagy-related cell death by modulation of the AMPK–mTOR–ULK1 signaling axis. Thus, our study established pharmacological sensitivity by both β-catenin and Akt signaling axis for leading autophagy in transformed hepatocytes as p53-dependent. An excessive stimulation of autophagy can be lethal for cancer cells with different apoptotic thresholds and genetic background. Our results suggest that autophagy induction may serve as an efficient anticancer therapy for HCC. Developing novel autophagy-modulating strategies may identify new therapeutic avenues for effective drug treatment.

## Materials and methods

### Reagents

Commercially available antibodies to HRP-conjugated p53, cyclin D1, CDK2, TIM23, LC3, p62, phospho-p53 (Ser392 and Ser46), p19-ARF, cMyc, Caspase 8, DRAM1, and LAMP2 were purchased from Santa Cruz Biotechnology, CA. Phospho-MDM2 (Ser166 and Ser186), total MDM2, cleaved PARP, total PARP, pro-caspase 9, Caspase 3, phospho-Drp1 (Ser616), Beclin1, phospho-Ulk1 (Ser757), phospho-AMPKα (Thr172), phospho-Beclin1 (Ser15), and phospho-mTOR (Ser2481) were procured from Cell Signaling Technology, MA. HRP-conjugated antibody to actin, Mdivi1, Z-VAD-fmk, chloroquine, and FH535 were purchased from Sigma-Aldrich, MO. LAPTM4B was purchased from Novus Biologicals, CO. Commercially available AZD5363, rapamycin, and mTOR inhibitor (AZD8055) were procured from Cayman Chemicals, MI.

### Cell culture

Multiple passages of immortalized human hepatocytes (IHH) that generated a transformed and tumorigenic phenotype, named THH, generated in our laboratory^49–51^, was used in this study. In addition, HepG2 and Hep3B cell lines (American Type Culture Collection, USA), and Huh7.5 (kindly provided by Charles Rice, Rockefeller University, NY) were also used as representative transformed hepatocytes of HCC. Cell lines were cultured in DMEM (Gibco BRL, NY) supplemented with 10% FBS (Gibco BRL, NY) and 1% penicillin–streptomycin (Sigma-Aldrich, MO) antibiotic cocktail, and maintained at 37 °C in a humidified 5% CO_2_ incubator. The cell lines were routinely tested to rule out mycoplasma contamination using commercial Lonza MycoAlert™ Mycoplasma Detection kit.

### Cell viability assay

Approximately 1 × 10^5^ hepatic cells were plated on a 96-well plastic plate and allowed to attach overnight. Cells were exposed to various concentrations of the inhibitors for different time points. Cell viability was evaluated by reduction of tetrazolium using MTS (Promega, WI) following the supplier’s protocol. LDH cytotoxicity assay was analyzed using a kit for cell death (Thermo Fisher Scientific, IL). Cellular cytotoxicity was calculated as a percentage of the ratio of LDH release.

### Apoptosis assay

THH was treated with AZD5363 (5 µM), FH535 (5 µM), or in combination. Cells were trypsinized after 48 h of treatment and suspended in Annexin V binding buffer. Apoptotic effect on hepatocytes was measured with Annexin V fluorescein isothiocynate (FITC)/propidium iodide (PI) apoptosis detection kit (Thermo Fisher Scientific, IL). FACS Caliber flow cytometer (BD Bioscience) FL1 and FL3 channels were used to capture 10^6^ events per sample, and cellular apoptosis was measured using FlowJo 7.5 software.

### Plasmid and transfection

Hep3B cells were plated on 35-mm culture plate and transfected with plasmid expressing pcDNA3-p53 wild-type (Origene, MD) or empty vector construct (500 ng/plate) using Lipofectamine 2000 following the manufacturer’s instruction (Life Tech, IL) for transient transfection. Further, THH was similarly transfected with Beclin1 shRNA (Santa Cruz Bio, CA), and was selected with puromycin (200 ng/ml).

THH was stably transfected with mCherry-EGFP-LC3B plasmid (Addgene, MA); puromycin-resistant cells were selected and pooled for use within two to three passages. The cells were treated with inhibitors for autophagy flux analysis^52^.

### Western blot analysis

Transformed hepatocytes were treated with AZD5363 (5 µM), FH535 (5 µM), or in combination for 48 h. After treatment, cells were washed with PBS, lysed, and the proteins were resolved by SDS-PAGE for Western blot analysis. The nitrocellulose membrane was blocked with 4% nonfat dry milk and incubated with primary antibody overnight at 4 °C. The membrane was washed with TBST buffer and incubated with secondary antibody for 1 h at room temperature. The blot was developed by chemiluminescence using ECL kit (Thermo Fisher Scientific, IL). Cellular actin was detected using a specific antibody for comparison of the protein load in each lane.

### Immunofluorescence

THH was grown in 35-mm cell culture dish and treated with the inhibitors for 48 h. Cells were fixed with 3.7% formaldehyde, permeabilized using 0.2% Triton X-100, and blocked with 5% BSA in room temperature for 2 h. Cells were incubated with primary antibody to total p53 (Santa Cruz Biotechnology, CA) or phospho-Drp1 (Ser616) (Cell Signaling Technology, MA) and Mitotracker (Thermo Fisher Scientific, IL) overnight at 4 °C. After incubation, cells were stained with appropriate fluorescence-conjugated secondary antibody for 1 h at room temperature and treated with ProLong Gold antifade reagent containing DAPI (Invitrogen, CA) for 2 min. Stained cells were visualized by confocal microscopy (Leica).

### Statistical analysis

Each experiment was performed at least three times, and the data are shown as the mean. The error bars present standard deviation of the experimental results. The differences between the control and test conditions were evaluated by two-tailed unpaired *t* test using GraphPad Prism 7 (GraphPad Software, La Jolla, CA) statistical software. A difference in value of *P* < 0.05 was considered statistically significant.

## Supplimantary information

Supplimantary Figures
Supplimantary Figure 1
Supplimantary Figure 2
