# Supplementary figures and images for: A combination of AZD5363 and FH5363 induces lethal autophagy in transformed hepatocytes
Source: Cell Death Dis. 2020 Jul 17;11(7):540. doi: 10.1038/s41419-020-02741-1 (PMC7367822; doi:10.1038/s41419-020-02741-1)

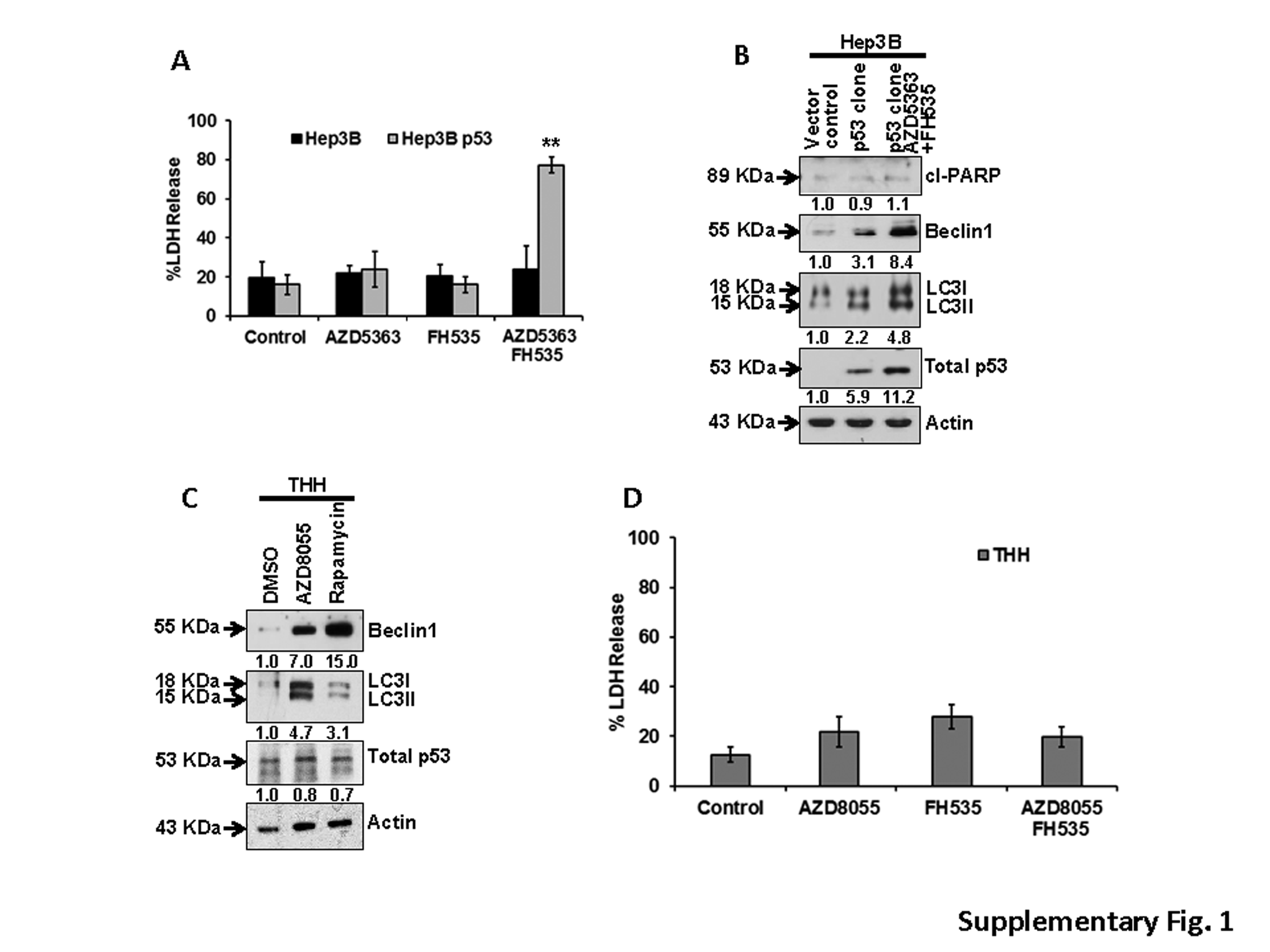

Supplement: Supplementary file 1 — Supplimantary Figures [file 41419_2020_2741_MOESM1_ESM.tif]

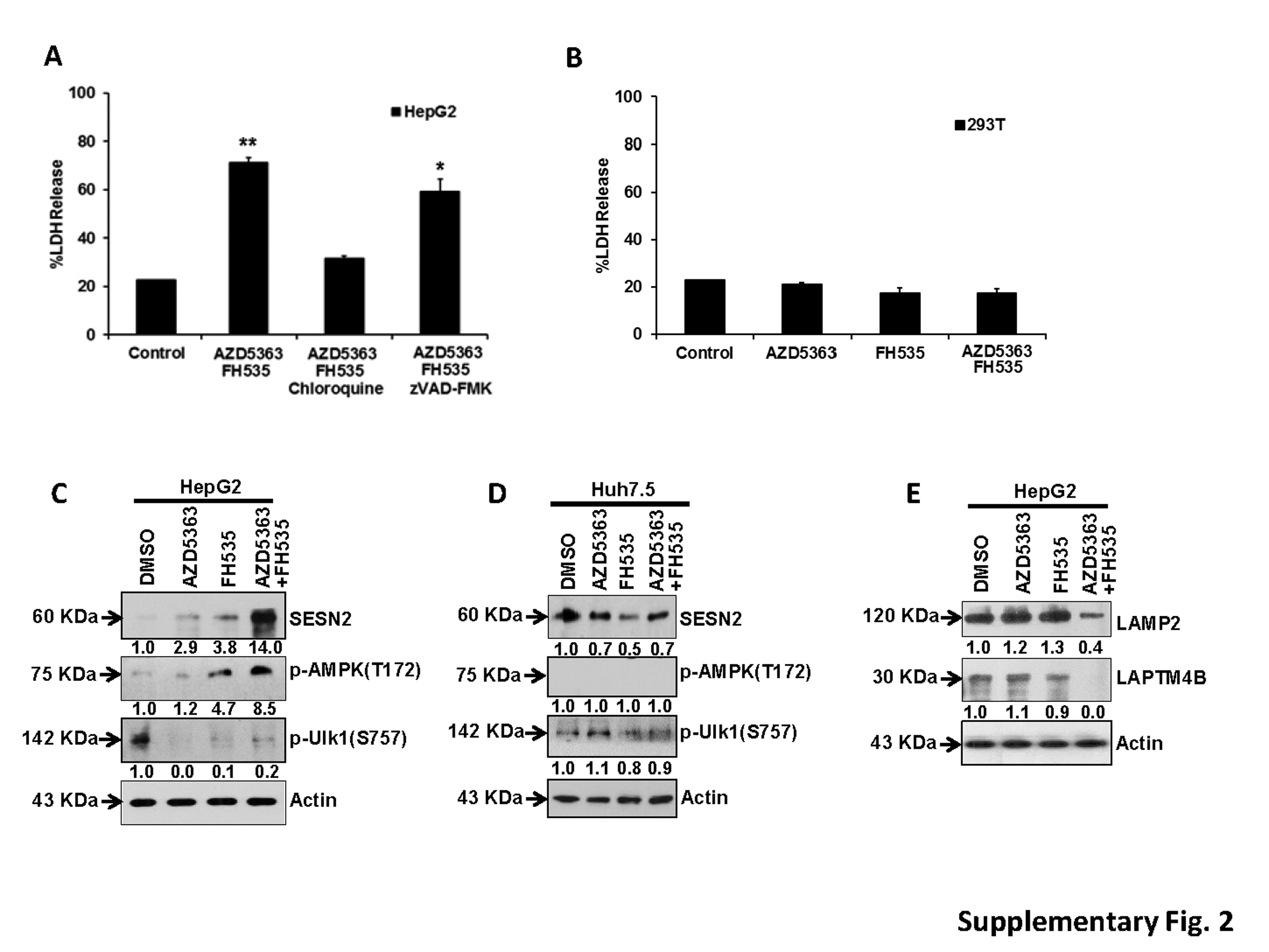

Supplement: Supplementary file 2 — Supplimantary Figure 1 [file 41419_2020_2741_MOESM2_ESM.tif]
