# Supplementary material for: A combination of AZD5363 and FH5363 induces lethal autophagy in transformed hepatocytes
Source: Cell Death Dis. 2020 Jul 17;11(7):540. doi: 10.1038/s41419-020-02741-1 (PMC7367822; doi:10.1038/s41419-020-02741-1)
Supplement: Supplementary file 3 — Supplimantary Figure 2 [file 41419_2020_2741_MOESM3_ESM.docx]

**Supplementary Fig 1. Regulation of autophagy marker protein expression in p53 transfected Hep3B cells following AZD5363 and FH535 treatment.** Hep3B and wild type p53 transfected into Hep3B cells were treated with individual or a combination of 5 µM FH535 and 5 µM AZD5363 for 72 h, and examined for cell death by LDH release assay (panel A). The results are presented the mean ± SD from three independent experiments. ***P*<0.005 was regarded as significant. Results from Western blot analysis for cl-PARP, Beclin1, LC3, and total p53 expression status using untreated Hep3B (vector control) and Hep3B transfected with wild type p53 experimental cells were treated with or without a combination of those inhibitors for 48 h are shown (panel B). The expression status of Beclin1, LC3 and total p53 by Western blot analysis after treatment of THH with vehicle control, 0.2 µM mTOR inhibitors AZD8055 or Rapamycin are shown (panel C). Expression level of actin in each lane of Western blots were considered for comparison of protein load and illustrated by representative blots shown at the bottom. Densitometry scanning results are shown below of each lane which normalized to the actin content and was expressed relative to controls set at 1.0. THH death was analyzed from LDH release at 72 h after individual or combined treatment of 0.2 µM mTOR inhibitor (AZD8055) and 5 µM FH535 (panel D). The results are presented the mean ± SD from three independent experiments.

**Supplementary Fig 2. Combination treatment of AZD5363 and FH535 leads to autophagy associated HepG2 cell death activating AMPK axis.** Cellular death was analyzed from LDH release assay at 72 h after combined treatment of HCC cell line HepG2 with 5 µM AZD5363 and 5µM FH535 in the presence of 25 µM chloroquine or 50 µM z-VAD-fmk (panel A). Human kidney derived 293T epithelial cells represent as non-tumor cells were treated with individual or a combination of 5 µM FH535 and 5 µM AZD5363 for 72 h, and examined for cell death by LDH release assay (panel B). The results are presented as the mean ± SD from three independent experiments. **P*< 0.05, ***P*<0.005 were regarded as significant. Western blot analysis was performed after treatment of HepG2 and Huh7.5 cells with individual or a combination of 5 µM AZD5363 and 5 µM FH535 inhibitors for 48 h. The expression status of SESN2, phospho-Ulk1 (Ser757) and phospho-AMPK (Thr172) are shown (panels C and D). The expression status of LAMP2 and LAPTM4B in HepG2 cell line are shown (panel E). Expression level of actin in each lane was considered for comparison of protein load and illustrated by representative blots shown at the bottom. Densitometry scanning results are shown below of each lane which normalized to the actin content and was expressed relative to controls set at 1.0.
